# Fluorescent Indolizine-β-Cyclodextrin Derivatives for the Detection of Volatile Organic Compounds

**DOI:** 10.3390/s8063689

**Published:** 2008-06-02

**Authors:** Matthieu Becuwe, David Landy, François Delattre, Francine Cazier, Sophie Fourmentin

**Affiliations:** Laboratoire de Synthèse Organique et Environnement (EA 2599), 145 Avenue Maurice Schumann, 59140 Dunkerque, France

**Keywords:** Indolizine-β-cyclodextrin, sensor, formation constants, docking, sensing capabilities, detection, Volatile Organic Compounds

## Abstract

This paper presents the synthesis, the structural determination and the sensing capabilities toward Volatile Organic Compounds (VOCs) of a new class of fluorescent indolizine-cyclodextrin sensors. Two different pathways, both involving bipyridinium ylides and 6-amino-b-cyclodextrin, have been used to carry out the synthesis of these sensors. The macrocycle structures were dominantly established by ^1^H-NMR spectra and systematically studied by molecular modelling (MM3, AM1, AM1-COSMO methods). The sensing capabilities of the sensors were evaluated by emission of fluorescence, during the inclusion of the guest (adamantanol or aromatic derivatives) into the cyclodextrin (CD) host cavity. The host/guest complex formation was investigated by formation constant determinations, using experimental methods, coupled with theoretical calculations of formation energies using a specific docking procedure. Both experimental and theoretical results suggest that some compounds would make very attractive sensors for VOC detection. Some compounds could also be taken into consideration as biological markers.

## 1. Introduction

**Figure 1.**
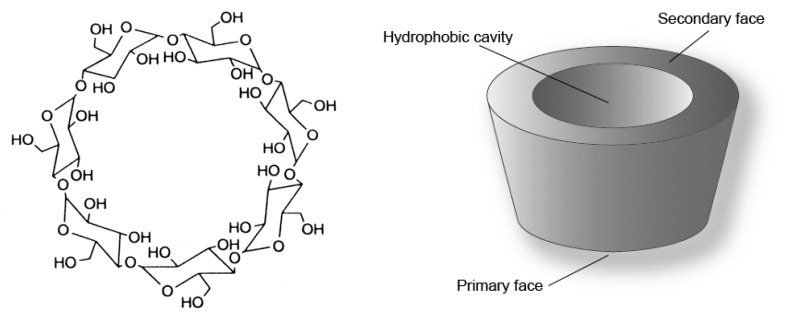
Schematic representation of β-cyclodextrin.

Cyclodextrins (CDs) are a family of cyclic oligosaccharides that are composed of α-1,4-linked glucopyranose subunits [1-3]. CDs are produced from starch by enzymatic degradation. The most common CDs are of three types: α-cyclodextrin (α-CD), β-cyclodextrin (β-CD) and γ-cyclodextrin (γ-CD), referred to as first generation or parent CDs (six, seven and eight glucosyl units, respectively). Among the parent CDs, β-CD is the most accessible, the lowest-priced and generally the most useful (see Figure 1) [4]. Chemically modified β-CDs with higher solubility than the first generation are commercially available.

CDs present a doughnut-like annular structure with wide and narrow hydrophilic tops delineated by O(2)H and O(3)H secondary and O(6)H primary hydroxyl groups respectively, and by a hydrophobic annular core lined with H(3), H(5) and H(6) hydrogen atoms and O(4) ether oxygen atoms. Generally, CDs can form host-guest complexes with a large variety of solid, liquid and gaseous organic compounds by a molecular inclusion phenomenon. This inner inclusion exerts a profound effect on the physicochemical properties of the guest molecules as they are temporarily locked or caged within the host cavity, giving rise to benefic modifications on the guest molecule properties (solubility, reactivity, volatility) [5]. That is why the native CD modifications are effective templates for generating wide ranges of molecular hosts [6].

Therefore, CDs are employed as carriers for biologically active substances [7], enzyme models [8], separating agents [9], catalysts [10], mass transfer promoters [11], additives in perfumes, cosmetics, aliments or food [12], environmental protection agents [13], or sensors for organic molecules [14].

CDs are essentially inert to photochemical excitation but their chemical modification with chromophoric moities may associate spectroscopic properties to the inclusion of guest molecule [15]. Therefore, they could be considered as biological markers or sensors for the detection of volatile organic compounds (VOCs).

This paper presents, in a synthetic way, our experimental and theoretical studies and results on a new class of fluorescent sensors, based on β-CD fragment bonded to some indolizine units conceived in our laboratory.

## 2. Results and Discussion

### 2.1. Synthesis and conformational study of the indolizine-β-cyclodextrin derivatives

The attachment of fluorophores to natural or synthetic receptors has received increased interest over last years in endeavours to furnish new fluorescent sensors. In particular, fluorescent CDs generated considerable interest among the synthetic community as witnessed some articles dealing on sensoring properties [16]. Alternatively, indolizine derivatives are relevant as biologically active products and are well known for exhibiting a variety of pharmacological effects, including cardiovascular, anti-inflammatory and antioxidant properties [17]. Besides, some indolizine compounds are also distinguished for their fluorescence properties. Some of them have already been used as dyes and biological markers [18]. That is the reason why we were gradually interested on the synthesis of new fluorescent sensors incorporating a fluorescent indolizine unit on the 6-amino-β-cyclodextrin fragment [19-21].

**Scheme 1.**
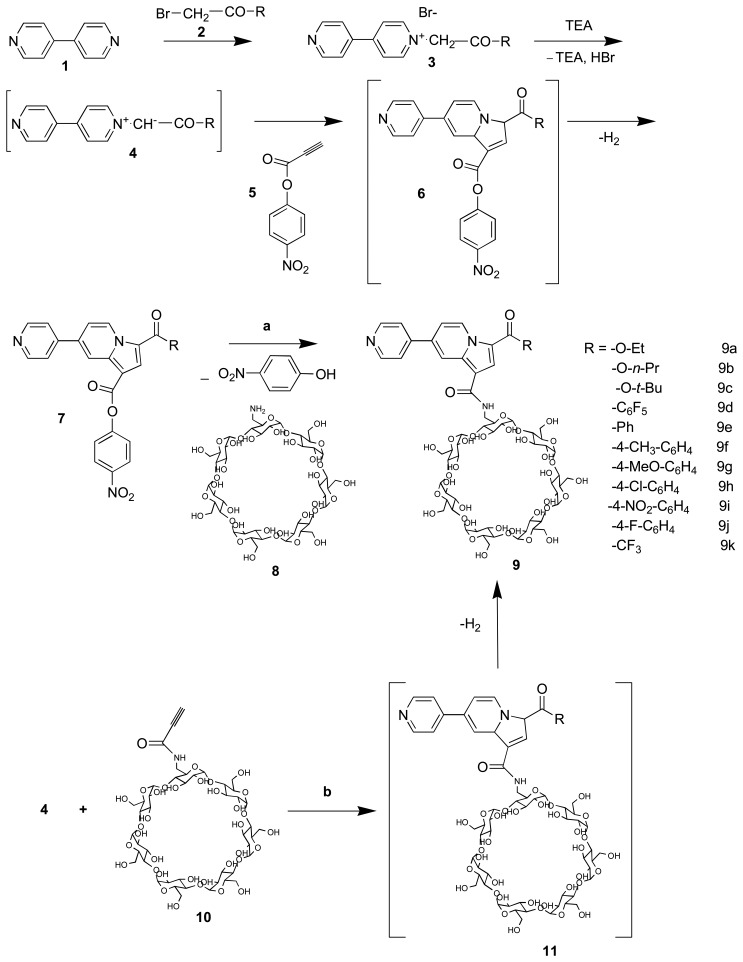
Synthesis of indolizine-β-cyclodextrin sensors.

The literature data offer several methods for the indolizine synthesis. Among them, the cycloaddition reactions involving cycloimmonium ylides and 1,3-dipolarophiles containing double or triple bonds were revealed as a highly effective and powerful strategy to build this heterocyclic scaffold [22]. Two different synthetic ways were employed (Scheme 1).

Briefly, the salt method [23] was applied to obtain the bipyridinium ylides **4**. The commercially available 1,4-bipyridine **1** is quaternized with active brominated organic derivatives **2**, forming the bipyridinium monosalts **3** with high yields. These salts form “in situ” in presence of triethylamine (TEA) the monosubstituted carbanion ylides **4**. These undergo a 1,3-dipolar cycloaddition reaction with 4-nitrophenylpropinoate **5**, to furnish primary dihydroindolizine cycloadducts **6**, which spontaneously provide the indolizine compounds **7** in good yields after a dehydrogenation reaction. However, the presence of the leaving group (4-NO_2_-Ph-O^-^) used for the first time in our experiments does not affects the yield compared to other dipolarophiles usually in use [24].

The mono-6-amino-6-deoxy-β-CD **8** was synthesized via a three steps pathway involving (i) a regioselective tosylation into the β-CD primary face, (ii) a substitution of the tosyl leaving group with NaN_3_ and (iii) azido group reduction using the Staudinger procedure [25]. Afterward, the mono-6-amino-6-deoxy-β-cyclodextrin **8** react with the indolizine derivatives **7** in homogenous conditions: dimethylformamide (DMF) or N-methylpyrrolidone (NMP), 50-60 °C, argon atmosphere, light absence during 18-24 hours, in order to provide the crude fluorescent β-CD sensors **9**.

In parallel, the identical sensors **9** were obtained by a 1,3-dipolar cycloaddition reaction connecting bipyridinium ylides **4** and propynamido-β-cyclodextrin **10** [26]. The primary cycloadducts **11** subsequently eliminates dihydrogen to furnish crude fluorescent indolizine-β-CD **9**. These reactions must be carried out without light, in order to prevent the cleavage of the ylide C^+^-N^-^ bond [27].

By both synthetic routes **a** and **b** (Scheme 1) the crude product **9** was isolated by precipitation from acetone and then successively purified using Sephadex CM-25 and G-15 chromatography. By comparing the yields for the eleven obtained derivatives, both synthetic ways **a** and **b** could be performed in accordance with the nature of the desired sensor. Systematically, the way **a** is recommended for the sensor **9** due to a better yield in all cases. Indeed, the leaving group *p*-NO_2_-Ph-O-is included in the macrocycle cavity inducing a favourable position for the coupling of the pyridinoindolizinic part. The synthetic yields of the compounds are between 25 and 35%.

**Scheme 2.**
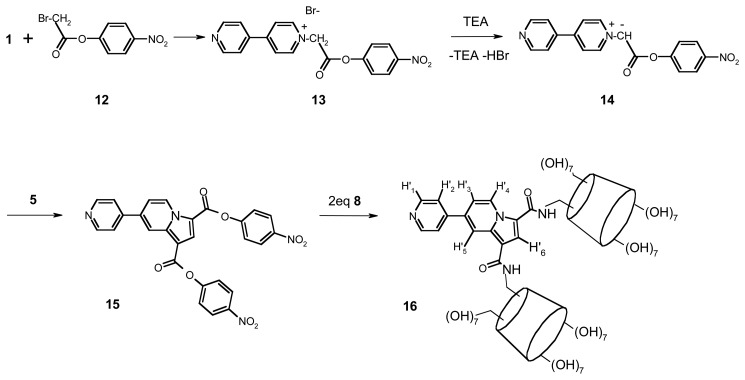
Synthetic pathway for the dimeric β-cyclodextrin sensor **16**.

Some other bis-CD sensors appear also in literature [28]. Thus, we have synthetised the 1,3-[bis-N-6A-deoxy-β-cyclodextrin-6A-yl-aminocarbonyl]-7-pyridin-4-yl indolizine (Scheme 2) as a part of our outgoing research program to develop a new range of fluorescent β-CD sensors [29].

To our knowledge, it is the first sensor containing in its structure two CD fragments linked to a fluorescent indolizine group.

**Figure 2.**
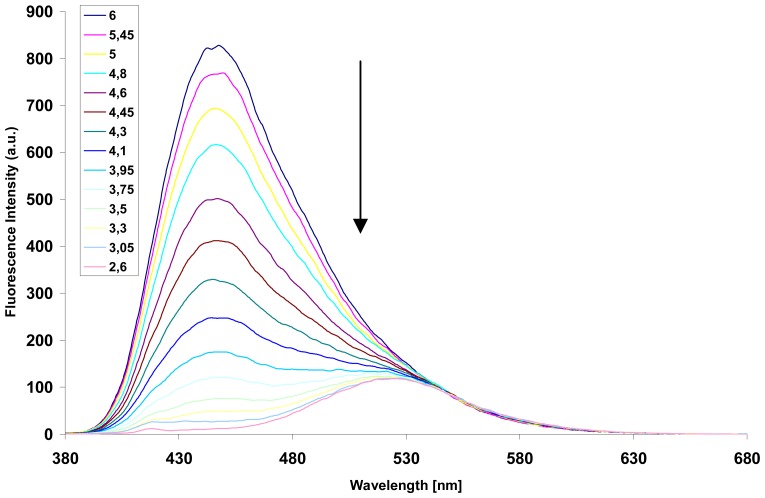
Reversible pH-dependence of the emission spectra of **9a** (0.0008mM, ^λ^_exc_.= 366 nm) in H_2_O at 25 °C.

Among these fluorescent molecular sensors, the compound **9a** was classified as a new pH-driven molecular switch [30] with a pKa value of 5.01. Indeed, it was shown that the decrease of pH into acidic domain leads to a drastic quenching of the fluorescence emission (figure 2) with a bathochromic displacement of the maximum emission.

**Scheme 3.**
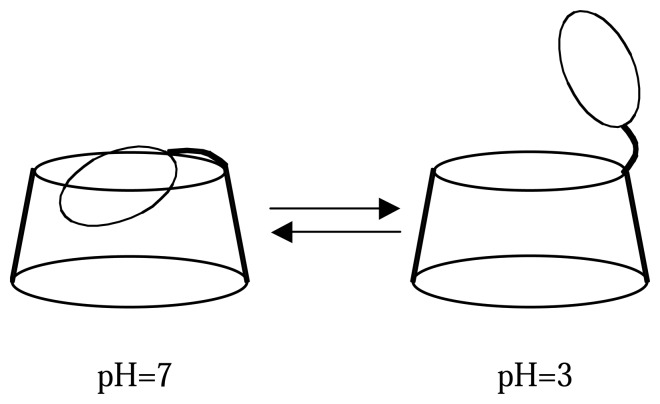
Structures of pyridin-4-yl indolizine β-cyclodextrin **9a** at pH 3 and pH 7.

To elucidate this phenomenom, we have carried out a 2D ROESY NMR experiments. It was clearly observed that spectrum recorded at neutral pH displays strong dipolar interactions between the protons localized inside the β-CD core and the aromatic protons of fluorophore. These through space interactions disappeared at acidic pH and reappeared following addition of alkaline deuterium solution. Thus these correlations clearly display the inside-outside molecular motion of fluorescent moiety, controlled by the protonation of free pyridyl nitrogen, inducing an extinction of fluorescence emission under acidic condition by exclusion of hydrophobic fluorescent moiety toward a bulk water environment (scheme 3).

All fluorescent β-CDs depicted in Scheme 1 and 2 have been characterized as sensors for some VOCs. Our strategy concerning the inclusion of VOCs on fluorescent indolizine sensors **9** and **16** was conducted in three steps: (i) the determination of the experimental formation constants of the sensor/VOC complexes using UV-Visible spectroscopy by either a direct titration or a spectral displacement method, (ii) the determination of sensor sensitivity towards 1-adamantanol and some representative aromatic VOCs and (iii) the calculation of the computed complexation energy by a specific molecular docking protocol [31].

### 2.2. Determination of the formation constant

Since the fluorescence sensitivity is depending on the fraction of the complexed sensor, we have first determined the inclusion ability of each sensor towards three guests: 1-adamantanol, because of its ability to bind strongly to β-CD, phenol and *p*-cresol, semi-volatile compounds which may be considered as the water soluble models for benzene and toluene VOCs. The determination of the formation constants has been realised by means of a spectrophotometric spectral displacement with methyl orange (MO) [32].

The complexation of MO by the CD cavity leads to a decrease of the absorbance, as a consequence of the encapsulation of the diazo part of the dye. Then, the addition of any guest implies a partial dissociation of the CD/MO complex, which results in an increase of the absorbance since a greater part of the MO is in free form. Such an increase is depending on the inclusion compound stability and on the guest total concentration. In addition, it has to be mentioned that the MO concentration and the observed wavelength range have been optimised to give the greatest variations upon inclusion with the various guests. Such variations then allow a precise determination of the formation constants, by means of a dedicated algorithmic treatment [33].

**Figure 3.**
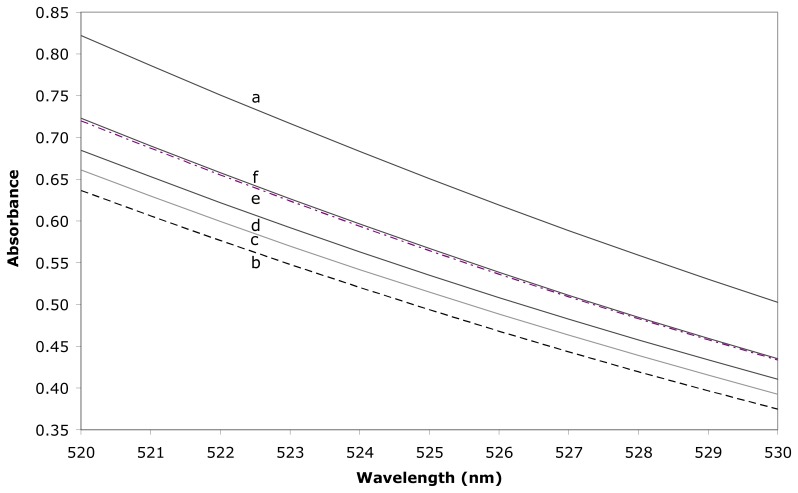
Absorption spectra (25 °C) for solutions containing (a) methyl orange 0.1mM, (b) methyl orange 0.1mM and Sensor **9j** 0.1mM, (c) methyl orange 0.1mM, Sensor **9j** 0.1mM and benzene 4.35mM, (d) methyl orange 0.1mM, Sensor 0.1mM **9j** and toluene 6.95mM, (e) methyl orange 0.1mM, Sensor **9j** 0.1mM and phenol 13mM and (f) methyl orange 0.1mM, Sensor **9j** 0.1mM and *p*-cresol 4.8mM.

An example of spectral variations is shown in Figure 3, in the case of sensor **9j**, which has been found as our most fluorescent sensitive modified cyclodextrin. The resulting formation constants are summarised in Table 1.

First of all, if one compares the genuine β-CD to the sensors, it seems that the inclusion compounds stabilities are very similar, leading to think that few steric interactions occurs between the various guests and the indolizine moiety of the sensor. This is in agreement with the so-called open cavity structures of the sensors, since the guest encapsulation does not imply any expulsion of the fluorescent moiety. In addition, and as could be expected from the β-CD inclusion ability, there is a strong difference of recognition between the aromatic guests and 1-adamantanol. While the stabilities are closed for phenol and *p*-cresol, 1-adamantanol leads to a significantly greater value of formation constant. Indeed, the 3-dimensional geometry of 1-adamantanol, if compared to the planar phenol and *p*-cresol, should lead to a greater filling of the internal cavity, thus increasing the Van der Waals stabilisation of the inclusion compound.

### 2.3. Determination of the sensing factors

**Figure 4.**
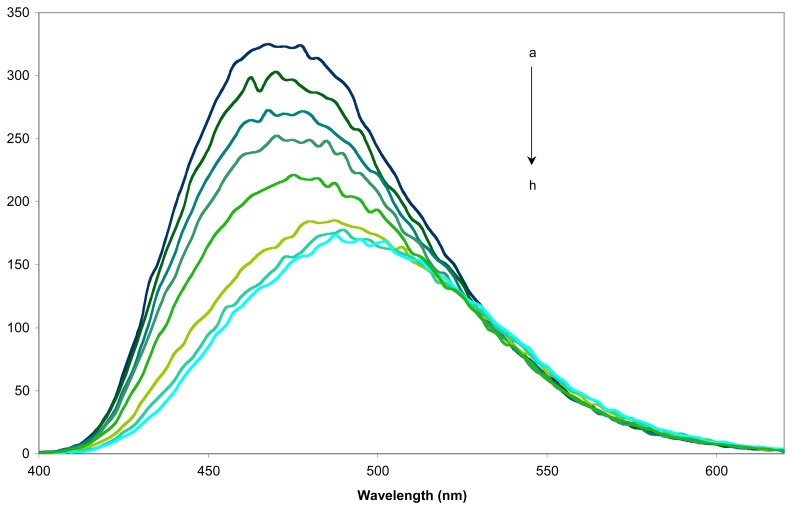
Fluorescence spectra of (a) the sensor **9j** in aqueous solution (0.08mM, 25 °C), at various concentrations of 1-adamantanol (b) 0.002mM, (c) 0.008mM, (d) 0.0125mM, (e) 0.02mM, (f) 0.05mM, (g) 0.08mM and (h) 0.2mM.

1-Adamantanol was chosen as guest to determine the sensing activity of the fluorescent sensors **9** and **16** for its ability to bind strongly into the inner cavity of β-CD and also for its non-fluorescent nature, which will not interfere with subsequent fluorescence measurements. An example of the fluorescence spectra obtained for sensor **9j** is given in figure 4.

The sensitivity factor ΔI/I_0_ was used to quantify the sensing abilities, where ΔI is I-I_0_, with I and I_0_ the emission intensities in the presence and absence of guest, respectively [34]. The obtained ΔI/Io values for the eleven sensors are summarized in Table 2.

Generally, the fluorescence spectrum of sensors **9** and **16** alone exhibit their fluorescent peak in the range of λ_max_ of 438-470nm, the excitation wavelength being between 274 and 370nm. By studying the quantitative data shown in Table 2 we find that two opposite behaviours of these indolizine sensors could be observed during the inclusion of the guest. The positive values of the sensing factors show an increasing of the emission intensity during the inclusion of 1-adamantanol in their inner cavity. In contrast, the negative values of the same factor suggest an emission intensity decrease during the inclusion. Nevertheless, the fluorescent indolizine sensors with little values of sensing factors could be considered as possible markers. The results obtained with these fluorescent indolizine sensors are comparable with those described in the literature for various fluorescent sensors containing β-CD and even greater for sensor **9e** and **9j** compared to the 6-O-dansyl-β-CD (ΔI/I_0_ = 0,39 for 1-adamantanol) [35].

We performed the same experiments for two semi-volatile compounds, phenol and *p*-cresol. The results obtained for some of the sensors are presented in Table 3.

**Figure 5.**
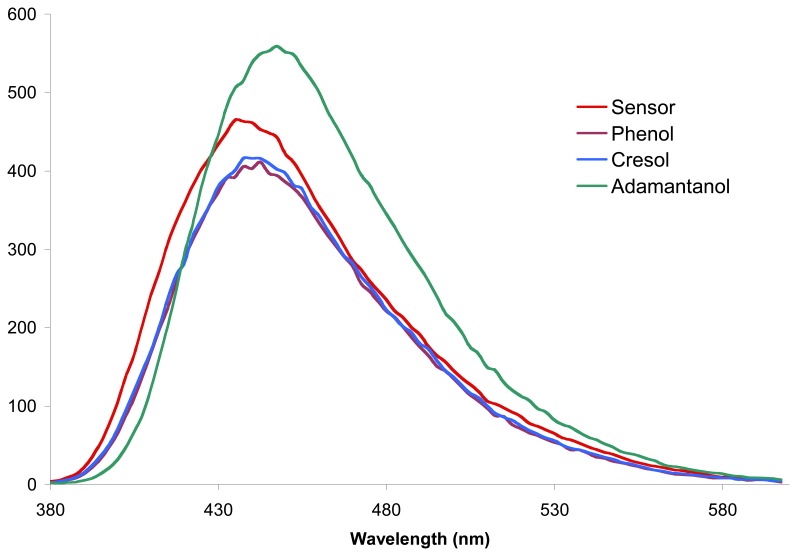
Fluorescence spectra of sensor **9c**, in aqueous solution (0.0002mM, 25 °C), in presence of 1-adamantanol 0.4mM, *p*-cresol 5mM and phenol 10mM.

As one can see, whatever the increase or decrease of the fluorescence emission intensity during the inclusion of 1-adamantanol, each sensor presents a decrease during the inclusion of the aromatic guests. Some bathochromic shifts upon inclusion of the guest have also been observed. As example, figure 5 depicts the fluorescence spectra of sensor **9c** in presence of the three studied guests.

**Figure 6.**
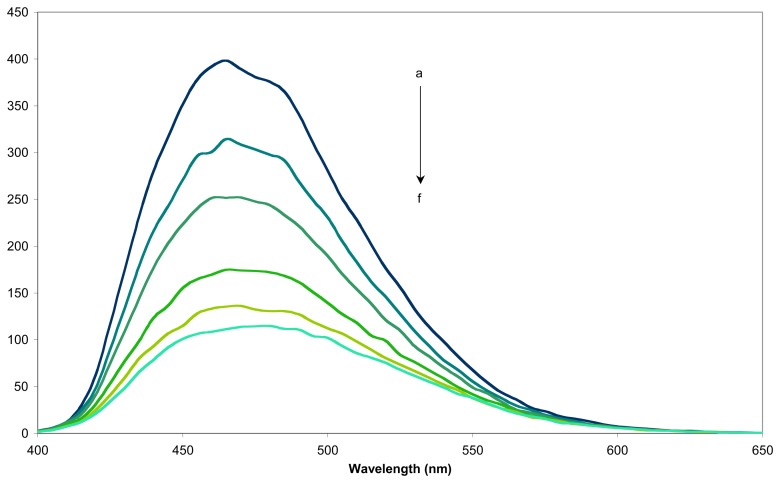
Fluorescence spectra of (a) the sensor **9j** in aqueous solution (0.1mM, 25 °C), at various concentrations of toluene (b) 2.7mM, (c) 5.4mM, (d) 13.5mM, (e) 18.9mM, (f) 27mM.

From these results we find that the most potent sensor in this class of compounds, is the N-6-deoxy-β-cyclodextrin-6-yl)-1-aminocarbonyl)-3-(4-fluorobenzoyl)-7-pyrridin-4-yl indolizine **9j** (see Scheme 1). So, we determine its sensing abilities for two VOCs, benzene and toluene. The obtained spectra are presented in Figure 6 in the case of toluene and the values of the sensitivity factors are given in Table 4.

The sensitivity factors obtained for the two VOCs are close to those of their semi-volatile analogous. This result can be explained because the only difference between these compounds is the hydroxyl group and validate the use of these compounds as models for our studies.

### 2.4. Molecular modelling

**Figure 7.**
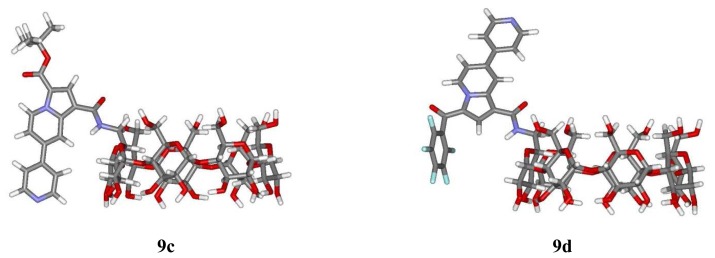
Structure of the most stable conformers of sensors **9c** and **9d**.

A multiconformational search has been realised on the sensors alone and has been followed by final geometry optimizations with two stages: a preliminary minimization with the MM3 method, and then an AM1 minimization without imposing any restrictions. Between the two general types of structures that could be identified, the open cavity type is more stable than its corresponding conformer with a capped cavity. The most stable structure orients the fluorescent moiety in such a way that few interactions occur with the CD cavity and that no self inclusion is observed (Figure 7).

It should be emphasised that the various dihedrals linking the CD to the fluorescent adduct are only constrained by the CD steric influence, so that the dynamical behaviour of such sensor should lead to the coexistence of many open conformations. The conformations thus only represent an instantaneous picture of the modified β-CD, but may show in what extent the sensors are able to make inclusion compounds with organic guests.

**Figure 8.**
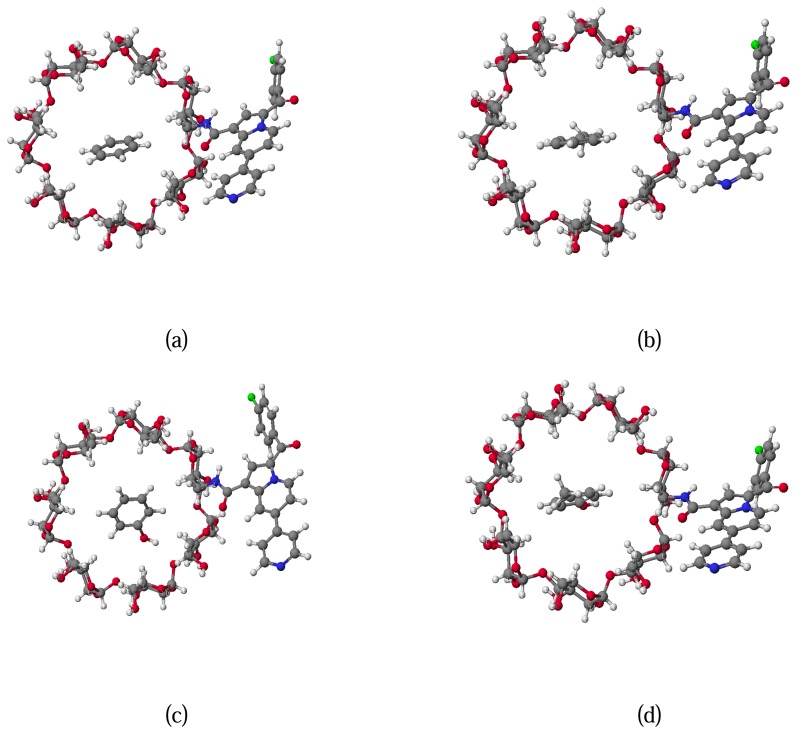
Representation of the top view of the conformation of host-guest complexes (a) benzene, (b) toluene, (c) phenol and (d) *p*-cresol with sensor **9j**.

Within this scope, we evaluated the energy gain upon association of the guest molecules (benzene, toluene, phenol and *p*-cresol) with the sensor **9j**, taking into account the 1:1 host-guest complex. In other words, we calculated the stabilization energy ΔE due to the inclusion of guest in the inner cavity of the sensor. The corresponding values and conformations are presented in Table 5 and Figure 8 respectively.

As the fluorescent moiety is kept outside of the cavity, there is no steric hindrance during the docking for any guest. This result explains that the formation constants observed for the sensors were closed to those of the genuine β-CD, the fluorescent adduct having little influence on the complexation. Moreover, since Van der Waals interactions are known to be the dominant part of the energetic stabilisation, the inclusion of toluene is predicted to be more stabilised than benzene, while cresol is better recognised than phenol. Both hydroxylated compounds also present more negative ΔE than their corresponding apolar species. Such results are in agreement with the experimental values, as the same order of stability is obtained. Since the four guests present similar structure, the entropic component of the complexation is likely to be proportional to the enthalpic part, in accordance with the enthalpy/entropy compensation. Thus, it is not surprising that the calculated enthalpic variation ΔE may be qualitatively correlated to the formation constants.

**Figure 9.**
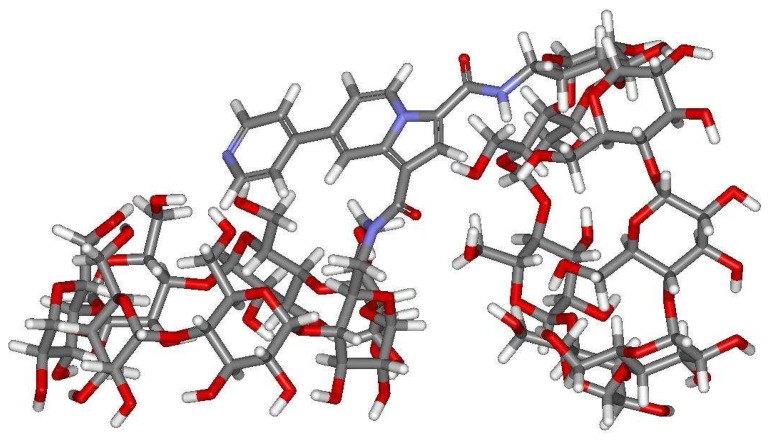
Predicted structure for the dimeric sensor **16**.

Sensor **16** was built like the other sensors from structural database, using the Cache library. The most stable structure was optimized after a multiconformational search based on MM3 force field, with the AM1 Hamiltonian in gaseous state and also in water. The most stable conformation is illustrated in Figure 9.

As can be observed, the bipyridine moiety of the indolizine fragment covers up the primary face of one of the two CDs frames. Moreover, the two cavities are directed in such a way that no cooperation could occur. Definitely, the inclusion of guest such phenol, *p*-cresol or 1-adamantanol requires the “face to face” conformation of the dimer to allow the simultaneous interactions of the guest with both cavities. Such results could explain the relatively poor efficiency observed in fluorescence detection: the sensing inclusion coefficients of phenol and *p*-cresol with sensor **16** are equal to -0.05 and -0.09 respectively.

We developed a new class of sensor for VOCs, by coupling the β-CD cavity with fluorescent indolizines. The sensing ability obtained for the most efficient of our compounds is among the highest observed in the CD field. As a consequence, we envisage immobilizing these CD sensors on solid support, in order to incorporate these compounds in portable badges for the detection of VOCs. In addition, some of our fluorescent CDs does not show such sensing ability and may thus be used as biological markers, since they maintain their inclusion ability.

## 3. Experimental Section

### 3.1 Chemicals

Benzene, toluene, phenol, *p*-cresol, 1-adamantanol, methyl orange, sodium hydroxide and potassium dihydrogenophosphate (Aldrich) were all of analytical reagent grade and were used as received. Deionised water was used throughout this work.

### 3.2. Fluorescence measurements

The measurements were carried out with a Perkin Elmer LS-50B fluorimeter at 25 °C and a quartz cell with an excitation angle of 90°. Excitation and emission slits were 4 nm. The absorbances of the studied guests are negligible at the excitation wavelengths used. The molar concentrations of the sensor in water range from 0.01mM to 0.00025mM. The emission spectra were recorded from 300 nm to 700 nm with a scan rate fixed to 120 nm/min. The control of temperature is realised by the use of a thermostated bath linked to the cell holder (accuracy: ± 0.1 °C).

### 3.3. Visible Spectra

Spectra were recorded using a Perkin Elmer Lambda 2S double beam spectrometer and a quartz cell with optical path length of 1.00 cm at 25 °C. All compounds were dissolved in phosphate buffer at pH 5.8. The control of temperature is realised by the use of a thermostated bath linked to the cell holder (accuracy: ± 0.1 °C). The stability of the complexes formed between MO and the hosts (sensors and β-CD) is first obtained by the use of the direct titration method; then, the complexing ability of the hosts is evaluated towards benzene, toluene, phenol, *p*-cresol and 1-adamantanol by means of a spectral displacement method with MO. Dedicated algorithmic treatments were applied to the first derivatives of UV spectra in order to avoid any spectral influence of diffraction phenomena [33]. Spectra were recorded between 520-530 nm for a MO concentration fixed at 0.1 mM. This wavelength range corresponds to the optimal spectral variation between the free and complexed forms of MO.

### 3.4. Molecular modelling

The sensor molecule was built starting from data provided by structural Database System of the Cambridge Crystallographic Data Center. The calculations were made using Spartan and Cache Libraries [36-37] on a PC computer. In order to obtain the most stable conformers for the sensors **9** and **16** we use a general procedure based on a MM3 multiconformational search [38]. This search consists in studying the ΔE potential energy variation according to the variation of different torsion angles by rotational increments of 15°.

**Figure 10.**
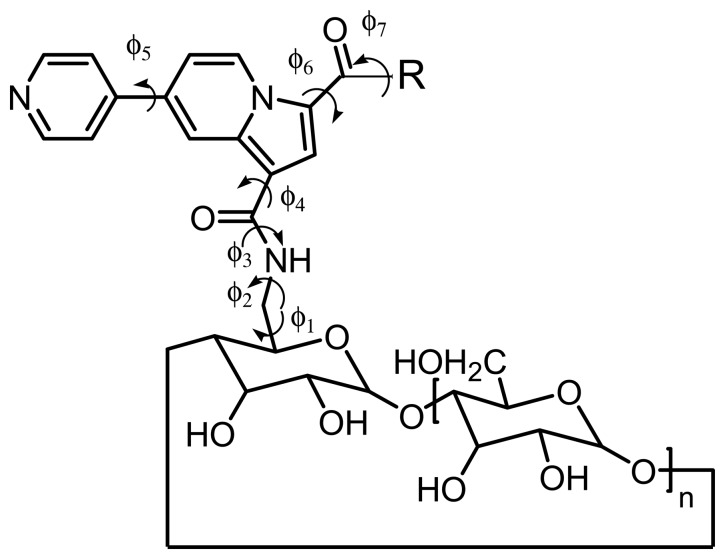
General structure of sensors **9**. ϕ_1_ to ϕ_7_ are the dihedrals controlling the sensor conformation.

The various torsion angles defining the sensor conformations are described in Figure 10. The rotation corresponding to dihedral angles ϕ7 and ϕ5 are not directly involved on our positioning of the fluorescent fragment in respect to the primary face of the β-CD fragment. The torsions according ϕ 1 and ϕ2 present a high proximity with the toroidal cycle of β-CD and consequently a reduced freedom. Thus, we may conclude that the two rotations described by ϕ3 and ϕ4 are adequate to locate the most stable conformations by systematic search. As consequence, only ϕ3 and ϕ4 are explicitly varied during the conformational search study, while ϕ1, ϕ2, ϕ5, ϕ6 and ϕ7 are only energy minimized. Once the minimum was obtained by this MM3 search, each conformation is freely minimized according to AM1 Hamiltonian in gas phase or in aqueous medium (COSMO solvent field).

### 3.5. Inclusion compounds conformation

The docking of each guest into the β-CD unit has been performed using four dummy atoms [31]. Each orientation has been taken in consideration for each guest. Three parameters were varied to explore the conformational space of the inclusion compound: the distance between host and guest, the orientation of the guest ring inside the host cavity, and its tilt angle. For this purpose, a sequential conformational search has been employed with the MM3 force field, with a systematic variation of each parameter. The most stables structures obtained by this procedure are then energy minimised without any constraint. The difference (ΔE, kcal/mol) between the energy of the inclusion complex and the sum of their individual components in their optimized ground states was then used as the theoretical parameter to evaluate the inclusion ability of sensor **9j**.

## Figures and Tables

**Table 1.** Formation constants (M^-1^) of the inclusion compounds formed between the sensors and various guests at 25 °C. Uncertainties are equal to ±10%.

	β-CD	Sensor **9c**	Sensor **9j**	Sensor **9k**
Benzene	82	55	53	46
Toluene	102	60	55	61
Phenol	115	122	85	110
*p*-Cresol	195	194	215	202
1-Adamantanol	34100	33800	33400	32100

**Table 2.** Sensitivity factors ΔI/Io towards 1-adamantanol, calculated for the maximum of emission of each sensor at pH 7 and [1-adamantanol]= 10* [sensor].

**Sensor**	**9a**	**9b**	**9c**	**9d**	**9e**	**9f**
ΔI/Io	+ 0.024	+ 0.110	+ 0.110	- 0.220	- 0.449	- 0.306
**Sensor**	**9g**	**9h**	**9j**	**9k**	**16**	

ΔI/Io	- 0.094	- 0.321	- 0.530	- 0.060	0.140	

**Table 3.** Sensitivity factors for four different sensors and the semi-volatile guests.

	ΔI/I_0_	
Sensor	Phenol	*p*-Cresol
**9b**	-0.07	-0.10
**9c**	-0.11	-0.10
**9d**	-0.23	-0.28
**9j**	-0.66	-0.65

**Table 4.** Sensitivity factors of sensor **9j** towards volatile organic compounds.

Guest	Benzene	Toluene
ΔI/Io	-0.54	-0.67

**Table 5.** Computed energy of complexation, ΔE (kcal/mol).

Guest	Benzene	Toluene	Phenol	*p*-Cresol
ΔE (kcal/mol)	-7.2	-8.9	-11.4	-12.7

## References

[1] Bender M.L., Komiyama M. (1978). Cyclodextrin chemistry.

[2] Szejtli J. (1988). Cyclodextrin technology.

[3] Szejtli J. (1998). Introduction and general overview of cyclodextrin chemistry. Chem. Rev..

[4] Szejtli J. (1997). Utilization of cyclodextrins in industrial products and processes. J. Mater. Chem..

[5] Singh M., Sharma R., Banerjee U.C. (2002). Biotechnological applications of cyclodextrins. Biotechnol. Adv..

[6] Szejtli J. (1982). Cyclodextrins and their Inclusion Complexes.

[7] Uekama K. (2002). Recent aspects of pharmaceutical application of cyclodextrins. J. Incl. Phenom. Macro..

[8] Fernandez M., Fragoso A., Cao R., Villalonga R. (2005). Stabilization of α-chymotrypsin by chemical modification with monoamine cyclodextrin. Process Biochem..

[9] Lelievre F., Yan C., Zare R.N., Gareil P. (1996). Capillary electrochromatography: Operating characteristics and enantiomeric separations. J. Chromatography A.

[10] Bhosale S.V. (2007). β-cyclodextrin as supramolecular catalyst in organic synthesis. Synlett.

[11] Blach P., Landy D., Fourmentin S., Surpateanu G., Bricout H., Ponchel A., Hapiot F., Monflier E. (2005). Sulfobutyl ether-β-cyclodextrins: Promising supramolecular carriers for aqueous organometallic catalysis. Adv. Synth. Catal..

[12] Szente L., Szejtli J. (2004). Cyclodextrins as food ingredients. Trends Food Sci. Tech..

[13] Fourmentin S., Outirite M., Blach P., Landy D., Ponchel A., Monflier E., Surpateanu G. (2007). Solubilisation of chlorinated solvents by cyclodextrin derivatives. A study by static headspace gas chromatography and molecular modelling. J. Hazard. Mater..

[14] Hattori K., Takeuchi T., Ogata M., Takanohashi A., Mikuni K., Nakanishi K., Imata H. (2007). Detection of environmental chemicals by SPR assay using branched cyclodextrin as sensor ligand. J. Incl. Phenom. Macro..

[15] Yoshida A., Yamasaki T., Aoyagi T., Ueno A.A. (2001). Molecule detection sensor of modified cyclodextrin based on guest-responsive intramolecular fluorescence quenching. Heterocycles.

[16] Ueno A. (1996). Review: fluorescent cyclodextrins for molecule sensing. Supramol. Sci..

[17] Gundersen L.-L., Malterud K.E., Negussie A.H., Rise F., Teklu S., Ostby O.B. (2003). Indolizines as novel potent inhibitors of 15-Lipoxygenase. Bioorgan. Med. Chem..

[18] Sonnenschein H., Hennrich G., Resch-Genger U., Schulz B. (2000). Fluorescence and UV/Vis spectroscopic behaviour of novel biindolizines. Dyes Pigments.

[19] Becuwe M., Delattre F., Surpateanu G.G., Cazier F., Woisel P., Garcçon G., Shirali P., Surpateanu G. (2005). Synthesis of new fluorescent β-cyclodextrin sensor. Heterocycl. Commun..

[20] Delattre F., Woisel P., Surpateanu G., Cazier F., Blach P. (2005). 1-(4-Nitrophenoxycarbonyl)-7-pyridin-4-yl indolizine: A new versatile fluorescent building block. Application to the synthesis of a series of fluorescent β-cyclodextrins. Tetrahedron.

[21] Lungu N.C., Depret A., Delattre F., Surpateanu G.G., Cazier F., Woisel P., Shirali P., Surpateanu G. (2005). Synthesis of a new fluorinated fluorescent β-cyclodextrin sensor. J. Fluorine Chem..

[22] Surpateanu G., Catteau J.P., Karafiloglou P., Lablache- Combier A. (1976). Structure and reactivity of cycloimmonium ylides. Tetrahedron.

[23] Depature L., Surpateanu G. (2002). Synthesis and characterisation of extended -bonding systems in cycloimmonium ylides derived from the 4,4′-bipyridine. Heterocycles.

[24] Druta I.I., Andrei M.A., Aburel P.S. (1998). Synthesis of 5-(2′-pyridyl)-indolizines by the reaction of 2-(2′-pyridyl)-pyridinium-ylides with activated alkynes. Tetrahedron.

[25] Defaye J., Crouzy S., Evrard N., Law H. (1999). PCT Int..

[26] Delattre F., Woisel P., Surpateanu G., Bria M., Cazier F., Decock P. (2004). 1,3-Dipolar cycloaddition reaction of bipyridinium ylides with the propynamido-β-cyclodextrin. A regiospecific synthesis of a new class of fluorescent β-cyclodextrins. Tetrahedron.

[27] Lablache-Combier A., Surpateanu G. (1976). Formation d'une azepine par-reaction photochimique d'un ylure de cycloimmonium de type N^+^ --- C^-^. Tetrahedron Lett..

[28] Narita M., Mima S., Ogawa N., Hamada F. (2001). Fluorescent molecular sensory system based on bis pyrene-modified γ-cyclodextrin dimer for steroids and endocrine disruptors. Anal. Sci..

[29] Surpateanu G.G., Landy D., Lungu N.C., Fourmentin S., Surpateanu G. (2007). New fluorescent bis-beta-cyclodextrin-indolizine sensor. Synthesis and sensing ability. J. Heterocycl. Chem..

[30] Becuwe M., Cazier F., Bria M., Woisel P., Delattre F. (2007). Tuneable fluorescent marker appended to β-cyclodextrin: a pH-driven molecular switch. Tetrahedron Lett..

[31] Decock G., Fourmentin S., Landy D., Surpateanu G.G., Decock P., Surpateanu G. (2006). Theoretical study on the inclusion of allergens with β-cyclodextrin and randomly-methylated-β-cyclodextrin. Internet Electron. J. Mol. Des..

[32] Decock G., Fourmentin S., Surpateanu G.G., Landy D., Decock P., Surpateanu G. (2006). Experimental and theoretical study on the inclusion compounds of aroma components with β-cyclodextrins. Supramol. Chem..

[33] Landy D., Fourmentin S., Salome M., Surpateanu G. (2000). Analytical improvement in measuring formation constants of inclusion complexes between β-cyclodextrin and phenolic compounds. J. Incl. Phenom. Macro..

[34] Narita M., Koshizaka S., Hamada F. (1999). Fluorescent pyrrolinone-modified cyclodextrins as a chemo-sensor for organic guests. J. Incl. Phenom. Macro..

[35] Ikeda H., Nakamura M., Ise N., Oguma N., Nakamura A., Ikeda T., Toda F., Ueno A. (1996). Fluorescent cyclodextrins for molecule sensing: fluorescent properties, nmr characterization, and inclusion phenomena of n-dansylleucine-modified cyclodextrins. J. Am. Chem. Soc..

[36] Spartan (2004). Version 4.0.

[37] CaChe (2003). Work System, 6.01.

[38] Surpateanu G.G., Vergoten G., Surpateanu G. (2000). A comparative study by AM1, PM3 and ab-initio HF/3-21G methods on the structure and reactivity of monosubstituted carbanion 1,2,4-triazolium ylides. J. Mol. Struct..

